# Effectiveness of INTI educational program on photoprotection among adolescents in high-altitude rural Andean school: A quasi-experimental study

**DOI:** 10.1016/j.puhip.2026.100853

**Published:** 2026-09-09

**Authors:** Yadhira Grecia Otazu Masco, Lizbeth Huarilloclla Ramos, Lucy Puño-Quispe

**Affiliations:** Universidad Peruana Unión, Faculty of Health Sciences, School of Nursing, Juliaca, Peru

**Keywords:** Solar radiation, Aging, Skin care, Health promotion

## Abstract

**Objective:**

To evaluate the effectiveness of an educational program (INTI) on knowledge, attitudes, and photoprotection habits among adolescents exposed to high levels of ultraviolet (UV) radiation in a high-altitude rural area of Peru.

**Study design:**

Quasi-experimental. Pretest-posttest study with non-randomized control and experimental groups.

**Methods:**

The study included 37 third-year secondary school students from a public school in the Andean region of Peru, who were assigned to a control group (n = 18) or an experimental group (n = 19) that received the 12-session INTI educational program. Knowledge, habits, and attitudes toward photoprotection were assessed before and after the intervention using the CHACES questionnaire. Between-group differences in posttest scores were examined using analysis of covariance (ANCOVA), adjusting for pretest score.

**Results:**

Pretest scores were similar between groups. At posttest, the experimental group showed higher mean scores than the control group in knowledge (*M = 6.58; M = 5.89*), habits (*M = 12.7; M = 10.4*), and attitudes (M = 33.2; M = 30.1). Analysis of covariance (ANCOVA) showed statistically significant between-group differences in knowledge (*p = 0.033*), habits (*p < 0.001*), and attitudes (*p = 0.019*) after adjusting for baseline scores.

**Conclusions:**

The study demonstrated that the INTI program significantly improved posttest scores for knowledge, habits, and attitudes related to photoprotection in the experimental group compared with the control group.

## 1 Introduction

Overexposure to ultraviolet (UV) radiation causes harmful damage to the health of animals and humans [1], with skin cancer being one of the most severe complications [2]. This occurs due to structural damage to deoxyribonucleic acid (DNA) caused by UV radiation; in response, the body's antioxidant mechanisms act to repair the damage. However, when these systems become saturated, the risk of carcinogenesis increases [3,4]. UV radiation can also cause significant damage to the skin structure and the immune system [5] and can cause ocular disorders such as cataracts, keratitis, and pterygium [6,7].

Prolonged exposure to ultraviolet radiation is associated with an increased risk [8], as UV radiation is a well-established carcinogen. At the global level, approximately 5400 people die each year as a result of this disease [9]. In Perú, the Ministry of Health reported 3525 cases of skin cancer between 2021 and 2023 [10]. Data from Peru's Ministry of Health indicate that malignant melanoma is the third most common cancer among individuals aged 15 to 39 years in the country. Although melanoma is recognized as the most aggressive and lethal form of skin cancer, it is also considered highly preventable, as an estimated nine in ten cases are attributable to ultraviolet (UV) radiation exposure [11]. In addition, skin cancer remains an underreported condition that disproportionately affects high-altitude Andean regions, where UV radiation travels a shorter atmospheric distance and reaches the skin with greater intensity, producing cumulative, often unnoticed damage. Unlike in coastal areas, where risk is tied to seasonal recreation, sun exposure in these regions is constant and embedded in daily life, particularly among outdoor workers, who often lack adequate protection because such exposure is normalized rather than perceived as a health risk [12]. The Andean region of Peru presents ultraviolet (UV) radiation levels classified as extremely high [13], a situation that is intensified by locations situated above 3, 800 m a.s.l. High-altitude conditions increase UV radiation penetration, thereby increasing the risk of skin lesions and triggering cumulative effects from an early age [14,15]. Children and adolescents are a vulnerable population, as approximately 80% of solar damage occurs before age 18.

International and regional evidence indicates that school-based educational interventions can meaningfully improve photoprotection-related knowledge, attitudes, and behaviors among children and adolescents. Programs implemented in Asia, North America, and Europe have reported gains in sun-safety knowledge and protective practices among primary and secondary school students [[16], [17], [18]], and comparable improvements have been documented across Latin America, including studies conducted in Colombia and, within Peru, in Huánuco and Chiclayo [[19], [20], [21]]. Evidence specific to the Puno region spanning preschool and primary school populations, including interventions that engaged parents as intermediaries, has similarly shown gains in photoprotection knowledge and practices [22,23]. Despite national and international studies examining educational programs for photoprotection and sunburn prevention, research specifically focusing on adolescents from rural areas remains limited. This gap is particularly concerning given the high levels of UV radiation present in the Andean region of Peru and the persistent problem of skin burns, which significantly compromise skin health. Within this context, adolescents are often more exposed to sunlight due to school-related activities, agricultural work, and outdoor recreation, and face limited access to health information and health care services. For this reason, the promotion and prevention of solar-exposure-related skin care through the implementation of an educational program becomes essential. In this context, the objective of the present study was to determine the effectiveness of the educational program in promoting photoprotection and preventing sunburns among third-year secondary school students at a rural, high-altitude institution, to strengthen knowledge, habits, and a positive attitude toward sun exposure.

## 2 Methods

### 2.1 Study design

The study adopted a quantitative approach using a quasi-experimental design with an experimental and a control group, both of which were assessed before and after the intervention [24,25]. It followed a longitudinal design, as data were collected at two different time points using the same measures [26]. Given the limited student population in this rural, high-altitude institution, only two third-year sections were available; these intact sections were randomly assigned to the intervention and control conditions. Randomization occurred at the classroom level, with only one classroom per condition.

### 2.2 Participants

The sample consisted of 37 third-grade secondary school students enrolled in a public educational institution located in the Andean region of Peru. One third-grade secondary school classroom served as the control group, comprising 18 students, while another classroom served as the experimental group, comprising 19 students who received the proposed intervention. Both groups were assessed using pretest and posttest measurements.

A non-probabilistic convenience sampling method was employed, which involves selecting participants based on the research's criteria and accessibility [27]. Eligible participants were aged 13-17 years who were enrolled at the participating educational institution during the study period and who were enrolled in the third-year secondary school grade established for this research; enrollment further required parental or legal guardian informed consent and student informed assent, as well as completion of the initial pretest evaluation. Students were excluded from the final analysis if they did not complete the required pretest or posttest evaluation, attended fewer than 11 of the 1 INTI program sessions, or voluntarily withdrew from the study at any point during its development. All 37 enrolled students met the eligibility criteria and completed the study; no participants were excluded or withdrew, and the final analytic sample corresponds to the full enrolled cohort.

### 2.3 Instruments

To measure habits, attitudes, and knowledge related to photoprotection, the CHACES (Questionnaire for the Study of Habits, Attitudes, and Knowledge in photoprotection among the Young Adults Population), selected based on established criteria for data-gathering instrument selection [28], previously validated and adapted into Spanish by Blázquez-Sánchez [29], was administered. The instrument comprises four dimensions: demographic data, sun-exposure habits, sun-related attitudes, and knowledge of sunburn prevention. It includes dichotomous questions and Likert-scale items. Content validity was assessed first by a panel of seven expert judges (five medical doctors and two community health nurses), who evaluated each item for relevance, representativeness, and clarity, yielding an Aiken's V coefficient of 0.91. Based on this review, minor wording adjustments were made to the original CHACES items: no substantial modifications were made to item content or meaning. A pilot test was then conducted with 15 secondary school students who had characteristics similar to the study population and did not form part of the final sample. This pilot test assessed item comprehension and provided a preliminary estimate of the instrument's reliability, yielding a Cronbach's alpha of 0.73. Given the limited size of this pilot sample, this estimate should be considered preliminary rather than a definitive indicator of reliability. Both coefficients were calculated by the research team for the present study.

### 2.4 INTI educational program

The INTI educational program, aimed at preventing sunburn among adolescents, consisted of 12 progressively structured educational sessions. The activities were delivered in person to third-grade secondary school students to promote photoprotective behaviors and skin health care. The sessions sequentially addressed the following topics: sun exposure, skin color, skin phototype, sunburn, sun exposure habits, sun protection practices, attitudes toward sun exposure, use of sunscreen, emotions and their relationship with the skin, the importance of preventing skin lesions from early ages, nutrition and hydration, and legislation and regulations related to sun protection.

The program was implemented over two months, with each session lasting 45 min. Evaluation was conducted before and after the educational intervention to assess changes resulting from the program's implementation, in accordance with recommendations in the literature [30]. In addition, the sessions were conducted using the ARDE methodology, structured into four stages: animation, reflection, demonstration, and evaluation [31]. It was considered that the adoption of healthy habits typically requires approximately two to five months for consolidation [32], which supports the duration of the implemented program.

### 2.5 Statistical analysis

Data were initially organized and entered in Microsoft Excel [33] before being imported into Jamovi software 2.3.24 [34]. For analysis, sample characteristics and study variables were summarized using frequency distributions, means, and standard deviations (SDs), which describe the variability of scores within each group [35]. Independent-samples t-tests compared pretest scores between the control and experimental groups for each dimension (knowledge, habits, and attitudes) to assess baseline comparability before fitting the ANCOVA models. Homogeneity of variances between groups was assessed using Levene's test for each dimension; normality of residuals was assessed using the Shapiro-Wilk test and Q-Q plots [36]; linearity between each covariate pretest score and its corresponding dependent variable posttest score was examined, along with homogeneity of regression slopes across groups. For each dimension, an ANCOVA was fitted with group as the fixed factor, posttest score as the dependent variable, and pretest score as the covariate, to estimate adjusted between-group differences in posttest score. Given the small sample size and the limited number of classrooms available for allocation, these assumption checks should not be interpreted as ensuring a fully valid and robust basis for inference. Rather, the findings are presented as adjusted between-group differences within a conventional ANCOVA framework, with corresponding caution regarding the strength of causal inference and the generalizability of the results beyond this specific population [37,38].

### 2.6 Ethical considerations

The research study was reviewed and approved by the Ethical Committee under approval number 2025-CEB-FCS – UPeU-225. Informed consent was obtained from parents or legal guardians, along with assent from the students, before their participation. All collected data were handled under strict conditions of confidentiality and anonymity, in compliance with personal data protection regulations.

## 3 Results

No students were excluded; the final sample matches the enrolled group (n = 37). Table 1 shows that the sample consisted of students evenly distributed across both grade levels, with a predominance of female participants (59.5%) and of 14-year-olds (62.2%). Intermediate skin tone was the most frequently reported (64.9), and most participants reported experiencing mild to moderate sunburn after sun exposure. In addition, a substantial proportion reported at least one episode of sunburn during the previous year, indicating frequent exposure to solar risk.

**Table 1:** Characteristics of the study participants.

	Variables	n	%
Grade and section	3rd grade "A"	18	48,6
	3rd grade "B"	19	51,4
Gender	Female	22	59,5
	Male	15	40,5
Age (years)	13	1	2,7
	14	23	62,2
	15	12	32,4
	17	1	2,7
Natural skin color	Dark	3	8,1
	Intermediate	24	64,9
	Light	10	27,0
How does your skin react when exposed to midday sun for 1 h?	No sunburn the next day; intense tanning after one week.	14	37,8
	Mild sunburn the next day; slight tanning after one week.	16	43,2
	Painful sunburn the next day; slight tanning after one week.	6	16,2
	Painful sunburn the next day; no tanning after one week.	1	2,7
How many times have you experienced sunburn (redness and pain) in the last year?	None	13	35,1
	1-2 times	8	21,6
	3-5 times	7	18,9
	6-10 times	3	8,1
	More than 10 times	6	16,2

Note. The table describes physical characteristics associated with susceptibility to sun exposure in the study population.

Table 2 presents the pretest measurements, showing that the control and experimental groups displayed comparable values across the three assessed dimensions: knowledge (control: M = 5.44; experimental: M = 5.11), habits (control: M = 11.7; experimental: M = 10.7), and attitude (control: M = 30.2; experimental: M = 29.2). Independent-samples *t*-test confirmed no significant differences between groups at pretest for knowledge (t [35] = 0.62, p = 0.538, d = 0.21), habits (t [35] = 0.75, p = 0.458, d = 0.25), and attitude (t [35] = 0.55, p = 0.584, d = 0.18), supporting an adequate level of baseline equivalence between groups before to the intervention. In the posttest assessment, the experimental group achieved a higher mean score than the control group in knowledge (M = 6.58; M = 5.89), habits (M = 12.7; M = 10.4), and attitude (M = 33.2; M = 30.1). In addition, lower dispersion was observed in the experimental group, particularly for habits and attitudes; this observed difference in variability should be interpreted with caution, as no formal statistical comparison of variances between groups was conducted. These descriptive results provide a consistent empirical context for interpreting the inferential analyses.

**Table 2:** Descriptive statistics of knowledge scores in the pretest and posttest by group.

Dimension	group	n	mean	SD	SE
Knowledge (posttest)	Control	18	5.89	0.9	0.212
	Experimental	19	6.58	0.902	0.207
Knowledge (pretest)	Control	18	5.44	1.423	0.336
	Experimental	19	5.11	1.853	0.425
Habits (pretest)	Control	18	11.7	4.88	1.151
	Experimental	19	10.7	2.9	0.666
Attitudes (pretest)	Control	18	30.2	6.2	1.461
	Experimental	19	29.2	5.5	1.262
Habits (posttest)	Control	18	10.4	4.54	1.07
	Experimental	19	12.7	1.56	0.357
Attitudes (posttest)	Control	18	30.1	6.37	1.501
	Experimental	19	33.2	2.97	0.682

Note: SD = standard deviation, SE = standard error of the mean.

Table 3 presents the ANCOVA results for knowledge scores. After adjusting for pretest knowledge, posttest scores differed significantly between groups (F [1,34] = 4.93, p = 0.033, η^2^p = 0.127): the experimental group showed a higher adjusted mean (EMM = 6.57, SE = 0.208, 95% CI [6.14, 6.99]) than the control group (EMM = 5.90, SE = 0.214, 95% CI [5.47, 6.34]). Pretest knowledge was not significantly associated with posttest scores (*p* = 0.419): this reflects the covariate's limited predictive value in this model and is independent of the group effect reported above.

**Table 3:** Analysis of covariance (Ancova) of knowledge scores (pretest and posttest) by group.

Source	Sum of squares	df	mean square	F	p	η^2^p
Overall model	4.585	2	2.293	3.021	0.062	
Group	4.036	1	4.036	4.926	0.033	0.127
Pretest knowledge	0.549	1	0.549	0.670	0.419	0.019
Residuals	27.860	34	0.819			

Note: df = degrees of freedom; η^2^p = partial eta squared.

Table 4 presents the ANCOVA results for habit scores. After adjusting for pretest habits, adjusted posttest scores differed significantly between groups (F [1,34] = 20.3, p < 0.001, η^2^p = 0.374): the experimental group showed a higher adjusted mean (EMM = 13.1, SE = 0.457, 95% CI [12.14, 14.0]) than the control group (EMM = 10.1, SE = 0.469, 95% CI [9.14, 11.1]). Pretest habits were also significantly associated with posttest scores (F [1,34] = 66.2, p < 0.001, η^2^p = 0.661), indicating that baseline habits strongly predicted posttest habits; this covariate effect reflects a predictive association rather than evidence of intervention effectiveness, which is captured separately by the group effect above.

**Table 4:** Analysis of covariance (ANCOVA) of habit scores (pretest and posttest) by group.

Source	Sum of squares	df	Mean square	*F*	*p*	η^2^p
Overall model	340.4	2	170.18	39.3	< 0.001	
Group	80.0	1	80.02	20.3	< 0.001	0.374
Pretest habits	260.3	1	260.35	66.2	< 0.001	0.661
Residuals	133.8	34	3.93			

Note: df = degrees of freedom; η^2^p = partial eta squared.

Table 5 presents the ANCOVA results for attitude scores. After adjusting for baseline attitudes, adjusted posttest scores differed significantly between groups (*F* [1,34] = 6.03, *p* < 0.019; η^2^p = 0.151): the experimental group showed a higher adjusted mean (EMM = 33.4, SE = 1.00, 95% CI [34.4, 35.5]) than the control group (EMM = 29.9, SE = 1.03, 95% CI [27.8, 32.0]). Pretest attitude scores were also significantly associated with posttest outcomes (F (1.34) = 10.68, p = 0.002, η^2^p = 0.239), reflecting the covariate's predictive value rather than evidence of intervention effectiveness.

**Table 5:** Analysis of covariance Ancova of attitude scores (pretest and posttest) by group.

Source	Sum of squares	df	Mean square	F	*p*	η^2^p
Group	115	1	114.7	6.03	0.019	0.151
Pretest attitude	203	1	202.9	10.68	0.002	0.239
Residuals	646	34	19.0			

## 4 Discussion

Photoprotection education is generally recommended to begin early in life, emphasizing physical barriers such as protective clothing, hats, and sunglasses, along with appropriate sunscreen use, given that programs targeting children and adolescents have shown favorable associations with increased knowledge and behavior change [39,40]. Although many educational programs show immediate improvements in knowledge and attitudes toward sun protection, sustaining these behaviors over the long term remains challenging. Ongoing reinforcement and tailored interventions are necessary to maintain behavioral change [41]. In this study, the INTI program was associated with higher adjusted posttest scores in knowledge, habits, and attitudes in the experimental group compared with the control group. ANCOVA results indicated a significant adjusted difference in adjusted means associated with group membership after controlling for baseline levels, with the largest difference observed for habits. This difference appears related to the direction of change within each group: the control group's habits scores fell from pretest to posttest, while the experimental group's rose, and this divergence, not simply a difference in the pace of improvement, helps account for the size of the adjusted difference. Attitudes changed little in the control group, suggesting this dimension may be harder to shift without direct intervention, while knowledge rose in both groups, suggesting part of that gain may not depend on the program alone.

These findings suggest that the INTI program was associated with promoting photoprotection-related behavioral change among secondary school students. A plausible explanation for the larger difference observed in habits relates to the structure of the INTI program itself: several sessions were built around repeated practice and demonstration, following the reflection and demonstration stages of the ARDE methodology, which may be more directly linked to habit change than to the more cognitive knowledge domain. These findings are consistent with those reported in a study conducted with 106 sixth-grade students from a middle school, which found that a multicomponent intervention was associated with improved knowledge and attitudes toward sun safety [42,43], as well as evidence from university and professional populations linking greater photoprotection knowledge to specific demographic characteristics, such as female gender [44]. Systematic review evidence similarly indicates that most school-based photoprotection interventions are associated with improved knowledge and behaviors among children and adolescents [45].

These findings reinforce the relevance of educational programs such as INTI in promoting behavior changes during adolescence, a critical stage of growth and development. However, studies have reported gender differences in photoprotection habits and in the use of preventive measures, highlighting the need to incorporate intervention strategies tailored to specific characteristics [46]. Furthermore, the family environment appears to play an important role in the adoption of preventive behaviors: parental knowledge of photoprotection has been associated with children's photoprotective practices [47], and parent-targeted educational interventions have improved children's protective behaviors [48]. These findings suggest that future iterations of programs such as INTI could be strengthened by expanding educational content for parents and caregivers, particularly in rural high-altitude contexts where sun exposure is high and socioeconomic conditions may compound UV-related health risks.

Regarding sun protection habits, the improvement observed in the experimental group contrasts with findings from a study among agricultural workers, in which a majority were unaware of the risks of prolonged sun exposure and expressed reluctance to use sunscreen [49]. Other interventions using digital tools, such as a facial aging web application, combined with educational messaging, have achieved sustained behavior change over a three-month follow-up [50], while longer interventions using printed materials and feedback have reported sustained protective behaviors over extended follow-up periods. [51] However, the duration of follow-up does not appear to be consistently associated with greater reductions in sun exposure, suggesting that other contextual factors such as poverty and limited access to health services, which are especially prevalent in rural populations [52], may influence the consolidation of photoprotective habits.

Regarding attitudes toward sun exposure, the improvement observed in the experimental group is consistent with prior work reporting improved attitude levels following educational interventions, [53] and with evidence that structured counseling approaches, including in-person, telephone, and mail-based strategies, sometimes paired with incentives, can support attitudinal change toward photoprotection. [54] Together, these findings suggest that structured educational interventions may help build adolescents’ psychological readiness for photoprotection, though sustaining these attitudes over time likely requires ongoing reinforcement.

The implications of this study support the inclusion of photoprotection education programs within the school curriculum, incorporating not only knowledge components but also practical habits and attitudes, which are essential for the primary prevention of skin cancer and premature skin aging. Integrating photoprotection education from an early age may help establish behaviors that reduce harmful exposure to ultraviolet radiation throughout life. Moreover, the results suggest the importance of continuous reinforcement of educational content and the use of diverse teaching strategies. Promoting programs such as INTI may help reduce the future burden of skin cancer and skin aging in vulnerable populations living in regions with high solar radiation.

Limitations of this study should be considered when interpreting the results. Because randomization occurred at the classroom level, with only one classroom per condition, group membership remains confounded with classroom membership, and the assumption of independence among individual observations may not be fully satisfied. This constraint limits the strength of causal inference and the generalizability of the findings. In addition, a non-probabilistic sampling method was used, which limits the generalizability of the findings to other school populations. In addition, contextual variables were not assessed, such as economic availability, access to health services, family influence, or cultural norms.

In conclusion, the findings of this study suggest that participation in the INTI program was associated with higher adjusted posttest scores in knowledge, habits, and attitudes related to photoprotection among third-year secondary school students in the experimental group compared with the control group. These scores reflect self-reported measures collected shortly after the intervention; whether the observed differences represent sustained behavioral change, rather than a short-term response captured at a single posttest point, was not assessed and remains an open question for future research. Given the Quasi-experimental, classroom-level design and modest sample size, these findings should be interpreted as preliminary evidence rather than definitive proof of the program's effectiveness.

### 4.1 Study contribution

This study evaluates a school-based photoprotection intervention in a population largely absent from the existing literature: secondary school adolescents in a rural, high-altitude Andean setting. Previous studies on photoprotection education have concentrated mainly on urban or peri-urban primary school children; evidence on rural, high-altitude adolescents, a group facing markedly higher cumulative UV exposure, remains scarce. Evaluating INTI in this specific population addresses that gap.

## Informed consent

Informed consent was obtained from all participants included in the study.

## Implications for policy and practice

• Integrate preventive sun education in rural schools
• Promote access to UV protection products.
• Strengthen school-based dermatological health policies.
• Encourage training programs for teachers and healthcare staff
• Promote awareness-raising and communication strategies.

## Ethical statement

All participants included in the study provided informed consent and assent before participation. Ethical standards were ensured throughout the research process in accordance with the Code of Ethics 2025-CEB-FCS – UPeU-225.

## Authors’ contributions

All the authors contributed substantially to the writing, data collection, review, and final adaptation of the manuscript.

## Funding

This study did not receive external funding.

## Declaration of competing interest

The authors declare that they have no known competing financial interests or personal relationships that could have appeared to influence the work reported in this paper.
